# Supplementary material for: The jaw is a second-class lever in Pedetes capensis (Rodentia: Pedetidae)
Source: PeerJ. 2017 Aug 30;5:e3741. doi: 10.7717/peerj.3741 (PMC5581530; doi:10.7717/peerj.3741)
Supplement: Table S1 — Landmark numbers correspond to Fig. 2. Landmarks 11–28 recorded on both sides of the skull. [file peerj-05-3741-s001.pdf]

Table S1. Landmark descriptions. Landmark numbers correspond to Figure 2. Landmarks 11-28 recorded on both sides of the skull.

#	Landmark description
1	Anteriormost point on internasal suture
2	Midpoint on dorsal cranium between anterior roots of zygomatic arches
3	Midsagittal point between anterior margins of ascending processes of zygomatic arches
4	Midpoint on dorsal cranium between zygomatic processes of squamosal bones
5	Posteriormost point on dorsal midline
6	Midpoint between ventral margins of incisor alveoli
7	Midpoint between anterior margins of premolars
8	Posteriormost midline point on palate
9	Midpoint between posterior margins of pterygoid flanges
10	Ventral midline point on margin of foramen magnum
11	Dorsalmost point on incisal alveolar margin
12	Anteriormost point on naso-frontal suture
13	Anteriormost point on dorsal margin of anterior zygomatic root
14	Premaxillo-maxillary suture on lingual incisal margin in infraorbital fossa
15	Anterior extremity of masseteric fossa on zygomatic arch
16	Dorsalmost extremity of infraorbital fossa
17	Lingual margin of incisor on posterior border of infraorbital fossa
18	Lacrimo-jugal suture on anterior orbital margin
19	Dorsalmost point on margin of masseteric fossa on zygomatic arch
20	Lacrimo-frontal suture on anterior orbital margin
21	Foramen on lacrimo-frontal suture in orbital wall
22	Notch between dorsal process of maxilla in anterior orbital wall and M2 alveolus
23	Anteriormost point on margin of optic foramen
24	Anterior extremity of fossa for insertion of posterior masseter on zygomatic arch
25	Medialmost point on dorsal orbital margin
26	Jugo-squamosal suture on dorsal margin of zygomatic arch
27	Posteriormost point on medial margin of zygomatic process of the squamosal
28	Posteriormost point on dorsal orbital margin
